# Acromegalic Uteropathy: Specific Uterine Ultrasound Findings in Female Patients

**DOI:** 10.3390/diagnostics16060956

**Published:** 2026-03-23

**Authors:** Irina Stanoevich, Aurika Asanova, Svetlana Vorotnikova, Andrey Belov, Ekaterina Grezina, Yulia Fedorova, Ugljesa Stanojevic, Larisa Dzeranova, Ekaterina Pigarova, Galina Melnichenko, Natalya Mokrysheva

**Affiliations:** 1 Endocrinology Research Centre, 11 Dmitriya Ulyanova St., Moscow 117036, Russia; 2849143@gmail.com (I.S.); bra_svetix@list.ru (S.V.); grezina.ekaterina@endocrincentr.ru (E.G.); dzeranovalk@yandex.ru (L.D.); kpigarova@gmail.com (E.P.); melnichenko.galina@endocrincentr.ru (G.M.); mokrisheva.natalia@endocrincentr.ru (N.M.); 2 Regional Budgetary Healthcare Institution “Kursk Oblast Scientific and Clinical Center named after G. E. Ostroverkhov”, 1 Eliseeva St., Kislino Khutor, Kursky District 305524, Russia; andreyi.belov@mail.ru (A.B.); ugljesha@mail.ru (U.S.); 3 Faculty of Medicine, Pirogov Russian National Research Medical University, 1 Ostrovityanova St., Bldg. 6, Moscow 117997, Russia; julia.dubna@mail.ru

**Keywords:** acromegaly, uterine cervix, uterine morphology, pelvic ultrasound, connective tissue disease, adenomyosis, growth hormone, insulin-like growth factor-1

## Abstract

**Background/Objectives:** Acromegaly is a systemic connective tissue disease driven by chronic growth hormone (GH) and insulin-like growth factor-1 (IGF-1) excess; yet, the female reproductive tract—especially the extracellular matrix (ECM)-rich cervix—has been poorly studied. We aimed to compare uterine and cervical morphology in women with acromegaly versus healthy controls and a gynecologic disease comparator, testing the hypothesis of selective cervical hypertrophy. **Methods:** We performed a retrospective case–control study of reproductive-age women who underwent pelvic ultrasound: acromegaly (*n* = 33), healthy controls (*n* = 45), and adenomyosis without acromegaly (*n* = 44). Uterine body measurements were obtained by TAUS/TVUS; cervical biometry was performed transvaginally in all cases. Volumes were estimated using the ellipsoid formula, and a uterus-to-cervix (U:C) volume ratio was calculated. Group differences were analyzed with Mann–Whitney tests and Bonferroni correction. **Results:** A total of 122 women were included. Uterine body length, width, AP size, and volume did not differ between acromegaly and either comparison group (all p-values non-significant). In contrast, cervical length, width, AP thickness, and volume were significantly higher in acromegaly than in healthy controls, with a corresponding reduction in the U:C volume ratio, indicating disproportionate cervical enlargement. Compared with adenomyosis, women with acromegaly again showed larger cervical width, AP thickness, and volume, together with altered U:C indices, whereas cervical length did not differ, suggesting a pattern not explained by nonspecific pelvic pathology. **Conclusions:** Women with acromegaly demonstrate a distinct uterine phenotype characterized by selective cervical hypertrophy with preserved uterine corpus size—an ECM-centric “acromegalic uteropathy.” This noninvasive morphometric signature may have diagnostic and procedural relevance and warrants confirmation in prospective studies.

## 1. Introduction

Acromegaly is a rare, chronic systemic disorder caused by prolonged exposure to excess growth hormone (GH) and its downstream effector insulin-like growth factor-1 (IGF-1), most often resulting from a pituitary somatotroph adenoma. The resulting endocrine milieu drives progressive multisystem involvement—classically soft-tissue overgrowth, organomegaly, connective tissue remodeling, and contributes to a wide range of metabolic and cardiovascular comorbidities [1]. Despite the breadth of recognized sequelae, the female reproductive tract, particularly the uterus and cervix, has not been comprehensively characterized in acromegaly with available data limited to isolated case reports and small, heterogeneous series.

Two converging lines of contemporary evidence support targeted assessment of uterine and, especially, cervical morphometry in acromegaly. First, GH/IGF-1 signaling exerts potent trophic effects on fibroblasts and extracellular matrix (ECM) biology. In human pelvic floor models, IGF-1 enhances fibroblast growth and modulates collagen metabolism via MAPK and NF-κB pathways; parallel findings across connective tissue systems demonstrate that IGF-1 promotes matrix synthesis (notably collagen I) and influences tissue repair dynamics [2,3]. These matrix-directed actions provide a mechanistic rationale for stromal hypertrophy under conditions of sustained endocrine excess.

Second, the cervix is a predominantly ECM-based organ. Contemporary reviews of cervical biology emphasize that ECM accounts for the vast majority of cervical tissue mass (commonly estimated at ~85–90%), whereas cellular elements—including a relatively small smooth muscle fraction—compose the minority. Cervical dimensions and biomechanics are governed by collagen architecture, cross-linking, and glycosaminoglycan-mediated hydration [4]. In contrast, the uterine corpus is dominated by smooth muscle (myometrium) with a comparatively lower ECM fraction. From this contrast arises a biologically coherent expectation: chronic GH/IGF-1 excess should disproportionately influence collagen-rich, hydrophilic connective tissues such as the cervix, potentially more than the myometrial uterus.

Clinically, reproductive manifestations in acromegaly—menstrual irregularities, subfertility, and pregnancy-related considerations—are typically interpreted through endocrine mechanisms (hyperprolactinemia, hypogonadotropic hypogonadism, treatment effects). These explanations, however, do not preclude a structural contribution from GH/IGF-1-driven stromal remodeling within the uterus–cervix unit. The published gynecologic literature in acromegaly remains sparse and heterogeneous. A recent 2024 systematic review on uterine fibroids (leiomyomas) in acromegaly reported inconsistent findings across small observational studies and highlighted methodological and confounding issues, leaving unresolved questions regarding the somatotropic axis and uterine disease burden [5]. Notably, virtually no studies have prospectively or systematically quantified cervical size, despite its ECM-rich composition and theoretical susceptibility to GH/IGF-1 trophic effects.

Ultrasonography provides a pragmatic avenue to address this knowledge gap. Current practice guidelines recognize transvaginal (and, when indicated, transabdominal) sonography as a reproducible method for assessing cervical dimensions and detecting disease-related changes in length and caliber. Standardized technique (e.g., minimizing probe pressure, mid-sagittal alignment) measurement improves reliability, while ratio-based indices can help normalize inter-individual variation [6]. Although uterine sonographic metrics are well established in gynecologic imaging, disease-specific cervical reference patterns outside obstetric contexts remain underdeveloped—precisely where acromegaly may contribute a distinct morphological phenotype.

Accordingly, we conducted a retrospective case–control study to compare morphometric parameters of the uterine corpus and cervix in women with a confirmed acromegaly against two comparators: age-matched healthy controls and women with adenomyosis (without acromegaly). We systematically measured and contrasted uterine and cervical characteristics across cohorts, and our exploratory finding is biologically plausible given contemporary endocrine and tissue-engineering insights: (i) GH/IGF-1 augments fibroblast activity and ECM synthesis in human connective tissues; (ii) cervical structure and mechanics are governed primarily by collagen-rich ECM and tissue hydration; and (iii) matrix-dominant organs would be expected to manifest the stromal imprint of acromegaly more readily than muscle-dominant ones [2].

Defining whether acromegaly is associated with a reproducible, noninvasive cervical morphometric signature has meaningful clinical implications. Because cervical length, caliber, and stromal properties can affect the technical ease of transcervical procedures, defining a reproducible cervical morphometric phenotype may also have practical relevance for procedure planning in gynecology and reproductive medicine. A validated imaging pattern (e.g., enlarged cervix with preserved uterine body, altered uterus-to-cervix proportionality) could: (a) serve as an auxiliary marker of systemic GH/IGF-1 tissue effects; (b) inform planning for transcervical procedures in reproductive medicine and gynecology, where dimensions and viscoelasticity influence instrumentation and success; and (c) generate hypotheses about obstetric and pelvic floor outcomes that warrant prospective evaluation. By comparing women with acromegaly to both healthy controls and gynecologic disease cohorts, our study aims to distinguish a somatotropic, connective tissue-specific phenotype from nonspecific pelvic pathology.

In summary, by integrating current insights into GH/IGF-1-ECM biology and contemporary sonographic methodology, we test the hypothesis that acromegaly is characterized by a selective, ECM-driven cervical hypertrophy—an underrecognized visceral manifestation with potential diagnostic and procedural relevance.

## 2. Materials and Methods

We performed a retrospective case–control study including three cohorts drawn from reproductive-age women with available pelvic ultrasound measurements: acromegaly (*n* = 33), defined by a confirmed diagnosis; healthy controls (*n* = 45), with no known endocrine disease; and adenomyosis (*n* = 44), with no history of acromegaly.

No formal a priori sample size calculation was performed, as this was a retrospective exploratory case–control study based on available clinical data. The final sample size therefore reflected the number of consecutive eligible patients with complete ultrasound datasets during the predefined enrollment period. Eligibility criteria are summarized in Table 1.

### 2.1. Ultrasound Acquisition

Measurements were obtained in orthogonal planes according to routine gynecologic ultrasound practice. The uterine body was evaluated in mid-sagittal and transverse views, while avoiding excessive probe pressure. As this was a retrospective study, uterine and cervical measurements were extracted from routine clinical ultrasound reports recorded at the time of examination; no centralized research-specific re-measurement was performed. All participants underwent a standardized pelvic ultrasound protocol at the study center that included both transabdominal (TAUS) and transvaginal (TVUS) examinations. Cervical biometry (length, width, and anteroposterior diameter) was performed transvaginally in all cases, with the endocervical canal centered; cervical length was measured from the external to the internal os, and width/AP diameter were recorded at their maximal orthogonal dimensions. No virgo patients were included, and TVUS was feasible for all participants; therefore, the ultrasound route for cervical measurements did not differ by cohort, and route-related differences in measurement sensitivity are unlikely to fully explain the between-group findings. All linear dimensions were recorded in centimeters. Uterine and cervical volumes were calculated using the ellipsoid formula: V = L × W × AP × 0.523 (≈π/6), where L = length, W = width, and AP = anteroposterior diameter; the same formula was applied to both the uterine body and the cervix. Ultrasound acquisition and morphometric variables used in the analysis are summarized in Table 2.

### 2.2. Statistical Analysis

Continuous variables were summarized as median [IQR]. Age is reported in decimal years. Overall three-group comparisons were assessed using the Kruskal–Wallis test. Because distributions were non-parametric, Mann–Whitney U tests were used for pairwise comparisons (acromegaly vs. controls; acromegaly vs. adenomyosis). Chi-square/Fisher’s exact tests were used for categorical data if applicable. Two-sided *p* < 0.05 (or *p* < 0.025 for Bonferroni-adjusted pairwise tests) indicated statistical significance. Analyses were performed on complete cases only.

## 3. Results

### 3.1. Cohort Overview

A total of 122 women were included: 33 with confirmed acromegaly, 45 healthy controls, and 44 with adenomyosis. Groups were age-matched by design. The pelvic ultrasound protocol was identical across cohorts (TAUS + TVUS for all participants), and cervical biometry was performed transvaginally in all cases.

### 3.2. Uterine Body Size and Volume

Across the three cohorts, no significant differences were observed in uterine body morphometry. Uterine length, width, anteroposterior (AP) dimension, and calculated uterine volume were statistically indistinguishable between women with acromegaly and both comparison groups (all *p*-values non-significant). Thus, within the predominantly myometrial uterine corpus, acromegaly did not demonstrate measurable enlargement under our imaging protocol.

### 3.3. Cervical Dimensions and Volume

In contrast, all three cardinal cervical measurements were significantly increased in the acromegaly cohort relative to healthy controls:

Cervical length: *p* < 0.0001.
Cervical width: *p* < 0.0001.
Cervical AP thickness: *p* < 0.0001.

These differences translated into a marked elevation of cervical volume in the acromegaly group (*p* < 0.0001). The consistency of findings across all measurement planes supports a global hypertrophic effect rather than a shape-specific artifact. Compared with controls, women with acromegaly demonstrated larger cervical linear dimensions and a higher cervical volume, while uterine body dimensions were comparatively less affected. This pattern yielded a consistently reduced uterus-to-cervix ratio, suggesting a preferential “cervical phenotype” rather than a diffuse enlargement of the whole uterus.

### 3.4. Proportionality of Uterus and Cervix

The uterine-to-cervical (U:C) volume ratio differed significantly between acromegaly and healthy controls (*p* = 0.001091). This ratio shift reflects a disproportionate cervical enlargement with relative preservation of uterine body size. Clinically, this disproportion may be more informative than any single absolute metric, as it normalizes for patient-level body size and inter-scanner variation. Descriptive statistics for all ultrasound parameters are summarized in Table 3.

### 3.5. Disease Comparator Analyses

To probe specificity, we compared acromegaly to women with adenomyosis. Compared with adenomyosis, acromegaly was associated with greater cervical width, AP thickness, and cervical volume (all *p*-values remained statistically significant), whereas cervical length did not differ between these groups (*p* = 0.876624). The uterus-to-cervix volume ratio remained significantly altered in acromegaly (*p* = 0.000152). These findings suggest that the cervical phenotype is not a nonspecific byproduct of pelvic pain disorders, chronic inflammation, or hormonal treatments typical of adenomyosis care, but rather a pattern distinctive of acromegaly-related connective tissue remodeling.

### 3.6. Sensitivity Considerations

The consistency of the results across the three orthogonal cervical axes argues against measurement bias from a single view. The significant ratio-based differences may reduce, but do not eliminate, concerns related to inter-individual variability. However, operator- and setting-related measurement variability cannot be excluded in this retrospective dataset (e.g., probe pressure, image optimization, and the absence of centralized re-measurement). As uterine body metrics did not differ across groups, the acromegaly-associated signal appears anatomically localized to the cervix rather than reflecting generalized uterine enlargement. Taken together, these data suggest the presence of a previously underrecognized acromegalic uteropathy characterized by disproportionate cervical hypertrophy with preserved uterine corpus dimensions.

## 4. Discussion

This study provides, to our knowledge, the first targeted ultrasonographic evidence that acromegaly in women is associated with selective cervical enlargement, while uterine body dimensions remain comparable to both healthy controls and a gynecologic disease comparator (adenomyosis). The consistent shift in internal proportionality (reduced U:C volume ratio) reinforces the concept that the cervix—rather than the uterine body—is preferentially involved.

**Figure 1:**
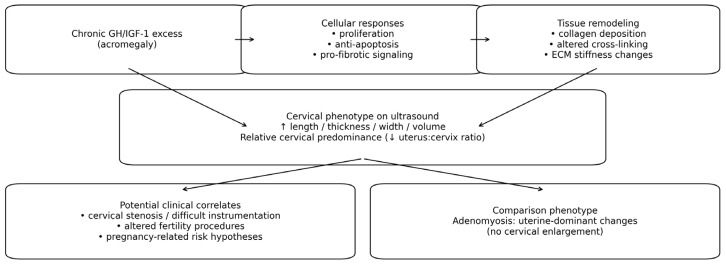
Proposed pathophysiological model of “acromegalic uteropathy” with preferential cervical stromal expansion.

The observed pattern is biologically coherent. The cervix is a matrix-dominant organ. As mentioned earlier, about 80–85% of its stroma is collagen-rich connective tissue, with a relatively small smooth muscle component. GH/IGF-1 signaling is known to stimulate fibroblast activation, enhance collagen synthesis, and expand ECM mass and hydration—mechanisms that underlie classic soft-tissue features of acromegaly (e.g., skin thickening, tendon enlargement, ligamentous hypertrophy) [7]. Contemporary structural and microstructural work confirms that cervical size, biomechanics, and viscoelasticity are governed by collagen architecture, cross-linking, and glycosaminoglycan-mediated hydration [8]. In a tissue whose bulk reflects ECM quantity and hydration more than muscle mass, sustained trophic drive from GH/IGF-1 would be expected to produce diffuse hypertrophy across all cervical axes—precisely the pattern observed here. By contrast, the uterine corpus is myometrium-predominant (smooth muscle), where size and stiffness are less directly dictated by collagen-rich ECM. If GH/IGF-1 excess preferentially augments fibroblast/ECM programs (via PI3K/AKT–MAPK pathways, among others), a matrix-heavy organ should exhibit the stromal imprint of acromegaly more readily than a muscle-heavy one [9]. The proposed pathophysiological model of “acromegalic uteropathy” with preferential cervical stromal expansion is summarized in Figure 1.

From a mechanistic standpoint, acromegaly is characterized by chronic exposure of connective tissues to GH/IGF-1 excess, which is known to activate stromal fibroblasts and to increase collagen turnover and extracellular matrix remodeling. A prospective biomarker study in active acromegaly reported higher circulating fibroblast activation protein (FAP) levels that declined after biochemical control, in parallel with markers of collagen turnover [10]. Together with broader consensus documents on acromegaly comorbidities and outcomes [11], these data support the biological plausibility that the cervix—an organ rich in collagenous stroma and proteoglycans—could enlarge predominantly through extracellular matrix expansion, altered hydration and cross-linking, and changes in vascular permeability.

Bioengineering studies further demonstrate that measurable cervical dimensions and mechanics are emergent properties of ECM microarchitecture and hydration, supporting the plausibility that sonographic enlargement reflects underlying ECM remodeling rather than mere geometric variation [12]. Broader translational literature also confirms IGF-1 as a key facilitator of type I collagen synthesis and ECM turnover [11]. ECM hydration driven by glycosaminoglycan content modulates tissue turgor and viscoelasticity and could further contribute to increased cervical volume [13].

Historically, gynecologic discussions in acromegaly focused on uterine fibroids, with inconsistent results likely driven by small samples and confounding issues [5]. Fibroids, as discrete smooth muscle tumors, do not model diffuse stromal responses, and our findings suggest that generalized uterine enlargement is not a prominent feature of acromegaly. Rather, the cervix appears to embody the connective tissue responsiveness of the somatotropic axis.

The comparison group with adenomyosis is informative: adenomyosis classically affects the uterine body and the junctional zone, with ultrasound definitions refined in recent years through the revised MUSA consensus [14]. Our data show that although adenomyosis may increase uterine volume, it does not reproduce the degree of cervical enlargement or the reduction in the uterus-to-cervix ratio seen in acromegaly. This distinction strengthens the concept of “acromegalic uteropathy” as a separate imaging phenotype and helps avoid misattribution of cervical changes to adenomyosis alone.

### 4.1. Potential Clinical Implications

First, a reproducible imaging signature (enlarged cervix with preserved uterine body; altered U:C ratio) might serve as an auxiliary marker of systemic disease activity or chronicity in women with acromegaly, analogous to heel-pad thickness or soft-tissue measurements elsewhere.

Second, these morphometric differences could be relevant for transcervical procedures (e.g., embryo transfer, endometrial sampling), where cervical caliber and stromal composition influence instrument passage and tissue mechanics. Cervical ECM content and hydration influence viscoelasticity and instrument passage; in other gynecologic contexts, sonographic cervical texture has been associated with procedure difficulty [15]. However, our study did not capture procedural, fertility, or obstetric outcomes; therefore, any clinical implications should be considered hypotheses for prospective validation.

Third, obstetric and urogynecologic sequelae are plausible but unproven. The balance between collagen content, cross-linking, and hydration determines cervical stiffness and compliance. If GH/IGF-1 excess increases collagen turnover and matrix mass, the net effect on competence in pregnancy (insufficiency vs. rigidity) is not self-evident and may depend on disease control and parity. Current reviews of pregnancy in acromegaly [16]) report generally reassuring maternal–fetal outcomes but highlight heterogeneous, observational data and a lack of standardized, cervix-specific phenotyping. In other words, current evidence says pregnancy is often safe overall, but it has not asked the question our data raise: whether acromegaly-related cervical enlargement—an ECM-centric phenotype—modifies risks relevant to pregnancy and delivery (e.g., cervical insufficiency vs. dystocia), or affects procedure planning in reproductive care [16]. Prospective registries should therefore pair GH/IGF-1 control with targeted cervical assessments (length/volume and uterus-to-cervix ratio by TVUS/3D, plus elastography or texture metrics) to define mechanics and outcomes; similar protocols could clarify potential urogynecologic implications (pelvic floor symptoms, lower urinary tract complaints, dyspareunia) in relation to cervical bulk and tissue properties. These hypotheses require targeted, longitudinal studies.

**Figure 2:**
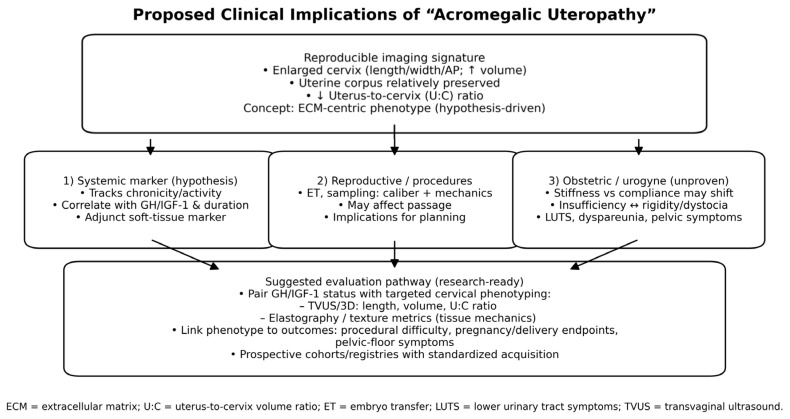
Proposed clinical implications of “acromegalic uteropathy” (cervical hypertrophy with preserved uterine corpus; altered U:C ratio).

Methodologically, our approach leveraged standard pelvic ultrasound and simple derived indices that are widely available and inexpensive. The use of ratio metrics (U:C volume) adds robustness by internal normalization. Ultrasonographic volume estimation by ellipsoid approximation remains routine in pelvic imaging, providing a pragmatic surrogate for true 3D anatomy [17]. Potential clinical implications of acromegalic uteropathy are summarized in Figure 2.

### 4.2. Future Directions: Standardized Imaging and Prospective Validation

Future work should standardize pelvic ultrasound acquisition and reporting using contemporary practice parameters and explicit measurement conventions (including ellipsoid-derived volumes), to improve reproducibility of cervical phenotyping across centers [18,19]. For differential diagnosis and comparator phenotyping, adenomyosis should be reported using revised MUSA definitions and complementary ratio-based ultrasound metrics (e.g., myometrial–cervical ratio), with emerging severity-grading approaches to support reproducible stratification [14,20,21,22]. Prospective registries could then pair GH/IGF-1 status (using contemporary consensus criteria and management guidance) with targeted cervical phenotyping and clinically meaningful endpoints, including technical outcomes of embryo transfer/other transcervical procedures and pregnancy-related outcomes, where current evidence is generally reassuring but heterogeneous [16,23,24,25,26,27,28,29,30].

### 4.3. Limitations and Strengths

Limitations include the retrospective design, complete-case analysis, and the lack of several key covariates that may influence pelvic morphometry, including parity, prior cervical procedures (e.g., conization/LEEP), hormonal therapy/contraception, menstrual cycle phase, BMI, fibroids and other uterine pathology, and detailed disease activity/duration in acromegaly (contemporaneous GH/IGF-1 levels, biochemical control status, treatment exposure, and time since diagnosis). Although a uniform pelvic ultrasound protocol was used at our center (TAUS + TVUS for all participants, with cervical biometry obtained transvaginally in all cases), the retrospective extraction from routine reports precluded centralized image review and research-grade metadata on operator identity/training, blinding, ultrasound settings, and inter-/intraobserver variability. We also did not correlate morphometry with contemporaneous GH/IGF-1 levels or disease duration, precluding dose–response inference.

Strengths include two comparator groups (healthy and disease), consistent directionality across axes, and the use of a proportionality metric (uterus-to-cervix volume ratio) as an internal normalization approach. The biological rationale is anchored in current GH/IGF-1–ECM science and modern characterization of cervical ECM.

## 5. Conclusions

In this retrospective ultrasound study, women with acromegaly demonstrated larger cervical dimensions and cervical volume with relatively preserved uterine corpus size compared with healthy controls. We propose that this pattern may reflect an ECM-centric connective tissue response to GH/IGF-1 excess (“acromegalic uteropathy”), but causality and clinical significance remain unproven. The next steps are to (i) validate these findings prospectively with standardized TVUS protocols and 3D/elastography measures; (ii) establish reference ranges for cervical volume and U:C across reproductive stages; (iii) correlate cervical morphometry with GH/IGF-1 control and disease chronicity; and (iv) assess clinically meaningful outcomes (e.g., procedures, pregnancy, and patient-reported outcomes).

## Figures and Tables

**Table 1:** Inclusion and exclusion criteria.

Domain	Criteria
Study design	Retrospective, single-center, cross-sectional ultrasound study.
Population	Women of reproductive age who underwent pelvic ultrasound (TAUS + TVUS; cervical biometry by TVUS).
Groups	Group 1: Confirmed acromegaly; Group 2: Controls without adenomyosis and without acromegaly; Group 3: Adenomyosis without acromegaly.
Inclusion (acromegaly)	Confirmed acromegaly (biochemical and pituitary imaging) and available pelvic ultrasound with complete uterine and cervical measurements.
Inclusion (adenomyosis)	Adenomyosis diagnosed by ultrasound using accepted morphological criteria; no biochemical evidence of acromegaly.
Inclusion (controls)	No ultrasound features of adenomyosis or other major uterine pathology; no clinical/biochemical evidence of acromegaly.
Exclusion	Pregnancy; history of hysterectomy; large uterine/cervical tumors distorting anatomy; prior pelvic radiotherapy; incomplete ultrasound dataset.

**Table 2:** Ultrasound acquisition and morphometric variables used in the analysis.

Domain	Variable/Definition	How Measured
Cervix	Cervical length (cm)	Sagittal view: internal to external os.
Cervix	Cervical AP thickness (cm)	Sagittal view at maximal thickness.
Cervix	Cervical width (cm)	Transverse view at maximal width.
Uterine body	Uterine length, width, AP diameter (cm)	Standard uterine measurements excluding cervix.
Uterine body	Uterine volume (cm^3^)	Ellipsoid formula: V = L × W × AP × 0.523 (≈π/6).
Ratios	Uterus-to-cervix volume ratio	Uterine volume/cervix volume.
Adenomyosis features	Direct and indirect MUSA features	Recorded according to revised MUSA definitions (see Section 4).

**Table 3:** Descriptive statistics. Note: *—Kruskal–Wallis test; **—Mann–Whitney test for pairwise comparisons (p1–2—Acromegaly vs. Controls; p1–3—Acromegaly vs. Adenomyosis; p2–3—Controls vs. Adenomyosis). Values are presented as median [interquartile range]. *p*-values below 0.0001 are reported as <0.0001. Bold is used for statistically significant *p*-values.

Parameters	Acromegaly (*n* = 33) Median [Q1; Q3]	Controls (*n* = 45) Median [Q1; Q3]	Adenomyosis (*n* = 44) Median [Q1; Q3]	*p* */*p* **
Age (years)	34.5 [31.0; 40.0]	32.8 [27.0; 39.0]	33.3 [29.0; 39.0]	*p* * = 0.4392
Uterine body, length (cm)	5.51 [4.8; 6.1]	5.19 [4.5; 5.8]	5.24 [4.75; 5.8]	*p* * = 0.1868; *p*_1–2_ = 0.091449; *p*_1–3_ = 0.146231; *p*_2–3_ = 0.792668.
Uterine body, width (cm)	4.97 [4.4; 5.5]	4.78 [4.0; 5.6]	4.96 [4.2; 5.7]	*p* * = 0.6298; *p*_1–2_ = 0.421769; *p*_1–3_ = 0.934518; *p*_2–3_ = 0.399706.
Uterine body, anteroposterior (cm)	4.04 [3.6; 4.6]	3.96 [3.3; 4.3]	4.26 [3.8; 4.95]	*p* * = 0.1586; *p*_1–2_ = 0.365044; *p*_1–3_ = 0.313281; *p*_2–3_ = 0.058246.
Uterine body volume (cm^3^)	54.37 [38.7; 66.12]	47.76 [31.36; 61.26]	52.91 [35.32; 62.46]	*p* * = 0.4630; *p*_1–2_ = 0.282533; *p*_1–3_ = 0.886624; *p*_2–3_ = 0.295345.
Cervical length (cm)	3.47 [3.2; 3.8]	3.05 [2.8; 3.3]	3.03 [2.7; 3.5]	*p* * = 0.0001; ***p*_1–2_ = <0.0001**; *p*_1–3_ = 0.876624; *p*_2–3_ = 0.701694.
Cervical thickness (AP) (cm)	2.86 [2.5; 3.1]	2.44 [2.2; 2.6]	2.45 [2.2; 2.75]	*p* * = <0.0001; ***p*_1–2_ = <0.0001; *p*_1–3_ = <0.0001**; *p*_2–3_ = 0.547696.
Cervical width (cm)	3.14 [2.8; 3.5]	2.63 [2.4; 2.9]	2.57 [2.1; 2.9]	*p* * = <0.0001; ***p*_1–2_ = <0.0001; *p*_1–3_ = 0.000126**; *p*_2–3_ = 0.914532.
Cervical volume (cm^3^)	17.17 [11.93; 21.44]	10.66 [7.92; 12.55]	10.5 [6.86; 13.31]	*p* * = <0.0001; ***p*_1–2_ = <0.0001; *p*_1–3_ = <0.0001**; *p*_2–3_ = 0.571198.
Uterus-to-cervix volume ratio	3.37 [2.17; 4.05]	4.89 [2.99; 6.08]	6.32 [3.5; 8.44]	*p* * = 0.0002; ***p*_1–2_ = 0.001091**; ***p*_1–3_ = 0.000152**; *p*_2–3_ = 0.295430.

## Data Availability

The datasets used and analyzed during this study are available from the corresponding author upon reasonable request.

## Abbreviations

The following abbreviations are used in this manuscript:

3D	three-dimensional
AKT	protein kinase B
AP	anteroposterior
ECM	extracellular matrix
GH	growth hormone
IGF-1	insulin-like growth factor-1
IQR	interquartile range
MAPK	mitogen-activated protein kinase
NF-κB	nuclear factor kappa B
PI3K	phosphoinositide 3-kinase
TVUS	transvaginal ultrasound
U:C	uterus-to-cervix
